# Obesity as a risk factor for severe influenza-like illness

**DOI:** 10.1111/irv.12156

**Published:** 2013-08-20

**Authors:** Noelle M Cocoros, Timothy L Lash, Alfred DeMaria, Michael Klompas

**Affiliations:** a Bureau of Infectious Disease, Massachusetts Department of Public HealthJamaica Plain, MA, USA; b Department of Epidemiology, Rollins School of Public Health, Emory UniversityAtlanta, GA, USA; c Department of Population Medicine, Harvard Medical School and Harvard Pilgrim Health Care InstituteBoston, MA, USA

**Keywords:** BMI, influenza, obesity

## Abstract

**Background:**

Obesity was recognized as in independent risk factor for influenza during the 2009 H1N1 influenza pandemic.

**Objectives:**

We evaluated the association between body mass index (BMI) and influenza-like illness (ILI) during two non-pandemic influenza seasons (2003–2004 and 2004–2005) and during the spring and fall waves of the 2009 H1N1 pandemic.

**Methods:**

Adults with severe (inpatient) and mild (outpatient) ILI were compared to those without ILI using a case-cohort design. The study was nested among those insured by a single health insurance company, receiving care from a large multispecialty practice. Data were collected from insurance claims and the electronic health record. The primary exposure was obesity (BMI ≥ 30·0 kg/m^2^).

**Results:**

Across three seasons, the crude and adjusted ORs for obesity and severe ILI were 1·65 (95% CI 1·31, 2·08) and 1·23 (95% CI 0·97, 1·57), respectively. An association was observed for those aged 20–59 years (adjusted OR 1·92, 95% CI 1·26, 2·90), but not for those 60 and older (adjusted OR 1·08, 95% CI 0·80, 1·46). The adjusted ORs for obesity and severe ILI in 2003–2004, 2004–2005, and during H1N1 were 1·14 (95% CI 0·80, 1·64), 1·24 (95% CI 0·86, 1·79), and 1·76 (95% CI 0·91, 3·42), respectively. Among those with a Charlson Comorbidity Index score of zero, the adjusted ORs for 2003–2004, 2004–2005, and H1N1 were 1·60 (95% CI 0·93, 2·76), 1·43 (95% CI 0·80, 2·56), and 1·90 (95% CI 0·68, 5·27), respectively.

**Conclusions:**

Our results suggest a small to moderate association between obesity and hospitalized ILI among adults.

## Introduction

Early investigations of 2009 pandemic influenza A H1N1 (2009 H1N1) noted that severity of illness was associated with obesity.^1^–^6^ Subsequent analyses suggested an association of obesity with both hospitalization and death from 2009 H1N1.^7^–^9^ Before the 2009 H1N1 pandemic, however, obesity was not recognized as an independent risk factor for influenza in general and severe influenza in particular.

Two groups have recently examined the association between body mass index (BMI) and influenza during non-pandemic influenza seasons. Coleman and co-authors conducted a case–control study of outpatient, laboratory-confirmed influenza during two typical flu seasons (2007–2008 and 2008–2009) and the 2009 pandemic season.^10^ They found no differences in mean body mass index (BMI) between cases and controls in each season. They interpreted their multivariate model as indicating no evidence of an association between obesity and influenza, although there was evidence – albeit not statistically significant – of increased odds of medically attended influenza among at least the highest category of BMI during the 2009 pandemic. Another group examined the association between obesity and respiratory hospitalizations in Ontario, Canada.^11^ These authors combined hospitalization claims data with self-reported BMI from 12 influenza seasons and found an association between obesity and hospitalized respiratory conditions among patients with and without recognized risk factors for severe influenza complications (i.e., chronic health conditions such as heart and respiratory disease).

Given these conflicting data, we examined the association between obesity and influenza-like illness (ILI) incidence and severity among adults during two non-pandemic influenza seasons (2003–2004 and 2004–2005) and during the spring and fall waves of the 2009 H1N1 pandemic. We hypothesized that obesity would be associated with increased risk for hospitalized ILI during non-pandemic influenza seasons as well as during pandemic H1N1.

## Methods

### Study population and data source

We compared individuals with mild (outpatient) and severe (inpatient) ILI to those without ILI using a case-cohort design, allowing us to use the same control group for both case definitions and individuals selected as a control may also be a case.^12^ We nested the study among individuals with Harvard Pilgrim Health Care insurance receiving care from Harvard Vanguard Medical Associates (HVMA), a large multispecialty ambulatory practice group based in Eastern Massachusetts. Cases, comorbidities, and outcomes were identified using insurance claims codes and electronic health record (EHR) data. We evaluated three study periods: two regular influenza seasons (2003–2004 and 2004–2005) and the pandemic H1N1 2009 season. The 2003–2004 season was chosen due to its relative severity in comparison with other recent seasons. The predominant influenza A strain during the 2003–2004 season was H3N2, although there was co-circulation of A(H1) and B. During the 2004–2005 season, A(H3N2) was also the predominant strain, but more B isolates were detected.^13^ The two non-pandemic seasons were defined as October 1, 2003, to May 31, 2004, and October 1, 2004, to May 31, 2005. The H1N1 pandemic period was defined as April 1, 2009, to December 15, 2009.

The following inclusion criteria applied to each cohort relative to the season's start date: age ≥ 20 years, no history of hospitalization in the prior year, no history of ILI in the preceding month, and measured BMI in the EHR during the study period or year preceding the start date. Pregnant women and underweight subjects (BMI < 18·5 kg/m^2^) were excluded.

Source data on body mass index, race/ethnicity, and temperature came from the electronic health record of the study practice. The practice uses the Epic Care (Epic Systems, Madison, WI, USA) electronic health record for all clinical transactions including registration, vital sign records, laboratory test ordering, laboratory test result tracking, and prescriptions. Clinicians assign diagnosis codes for each visit using dropdown menus. The dropdown menus are pre-populated with the diagnosis codes used most frequently by each practice, but clinicians have the freedom to select and assign any diagnosis code. Diagnosis codes for case determination were drawn from patients' insurance claims records.

### Case identification

Cases were identified on the basis of International Classification of Disease, 9th Revision, Clinical Modification (ICD-9) codes (Table 1) using a previously validated definition.^14^–^16^ Cases required at least one code from Table 1; if the EHR contained a measured temperature from the same encounter, it had to be >37·8 degrees Celsius for the case to be counted. Two types of ILI cases were identified: mild and severe ILI. Mild ILI was based on diagnostic codes from outpatient visits only, including emergency department visits. Severe ILI was based on diagnostic codes from inpatient visits. Severe ILI is the primary outcome of interest. Inpatient visits included all inpatient stays, same-day hospital discharges, hospital transfers, observation bed stays, and acute hospital care where the discharge was after the admission date. Cases who had an outpatient visit meeting the mild ILI case definition and then had an inpatient visit meeting the severe ILI definition during the study period were classified as severe cases. If there were multiple mild or multiple severe ILI visits, the case date was assigned based on the first such visit.

**Table 1:** ICD-9 codes used to identify mild and severe cases of influenza-like illness (ILI)

79·89	Viral infection, NEC	480	Viral pneumonia
79·99	Viral infection, NOS	480·8	Pneumonia due to virus NEC
460	Nasopharyngitis, acute	480·9	Viral pneumonia unspecified
461	Sinusitis, acute	481	Pneumococcal pneumonia
462	Pharyngitis, acute	482	Other bacterial pneumonia
463	Tonsillitis, acute	482·2	Pneumonia due to *Haemophilus influenzae*
464	Laryngitis and tracheitis	482·3	Pneumonia due to Streptococcus
464·01	Laryngitis, acute, with obstruction	482·4	Staphylococcus pneumonia NOS
464·1	Tracheitis, acute, without obstruction	482·41	MSSA pneumonia
464·11	Tracheitis, acute, with obstruction	482·49	Other staphylococcus pneumonia
464·2	Laryngotracheitis, acute, without obstruction	482·9	Bacterial pneumonia unspecified
464·21	Laryngotracheitis, acute, with obstruction	483	Pneumonia due to other specified organism
465	Acute upper respiratory infections of multiple or unspecified sites	484·8	Pneumonia in other infectious disease
464.2	Laryngotracheitis, acute	485	Bronchopneumonia, organism NOS
465·8	Infectious upper respiratory, multiple sites, acute NEC	486	Pneumonia, organism NOS
465·9	Infectious upper respiratory, multiple sites, acute NOS	487	Influenza
466	Acute bronchitis and bronchiolitis	487.0	Influenza with pneumonia
466.0	Bronchitis, acute	487·1	Influenza with respiratory manifestation NEC
466·1	Bronchiolitis, acute	487·8	Influenza with manifestation NEC
466·19	Bronchiolitis, acute, due to other infectious organism	784·1	Pain, throat
478·9	Disease, upper respiratory NEC/NOS	786·2	Cough

NOS, not otherwise specified; NEC, not elsewhere classified.

Cases had at least one of the respiratory codes listed, and a measured fever of at least 100F, an ICD-9 code of 780·6 (fever), or no measured temperature or ICD-9 code.

### Control selection

We randomly selected four times the number of controls as there were mild cases per cohort. These 4:1 controls were a random sample of the eligible participants. There was no matching of controls to cases on any factor.

### Exposure variable

Body mass index was obtained from the vital signs information in the EHR. We included values collected at any time during the study period or the preceding 12 months. For cases, we used BMI measured closest to the case date. For controls, we used the BMI measured closest in time to the season start date. It was acceptable to take height and weight from different visits to calculate BMI, if necessary, as long as both were within the defined timeframe.

The primary exposure of interest was obesity, defined as BMI ≥ 30·0 kg/m^2^ (WHO). We also examined weight categorized as overweight (BMI 25·0–29·9 kg/m^2^). Normal weight (BMI 18·5–24·9 kg/m^2^) was the reference category.

### Covariates

Charlson comorbidity index scores (Charlson scores) 17 were calculated based on ICD-9 codes documented in the EHR within 1 year of the season start date for the following conditions: myocardial infarction, congestive heart disease, peripheral vascular disorder, cerebrovascular disorder, dementia, chronic pulmonary disease, rheumatologic disease, peptic ulcer disease, mild liver disease, diabetes, diabetes with chronic complications, hemiplegia or paraplegia, renal disease, malignancy including leukemia and lymphoma, moderate or severe liver disease, metastatic solid tumor, and AIDS.

### Statistical analysis

We computed the frequency and proportion of subjects within BMI categories. We calculated odds ratios (OR), with corresponding 95% confidence intervals (CI), associating the occurrence of severe or mild ILI with overweight and obesity, relative to normal weight. We examined potential confounders in binary analyses and by change in the OR. We used multivariate logistic regression to adjust for likely confounders. Analyses were performed using sas version 9·3 (SAS Institute, Inc., Cary, NC, USA).

This study was approved by the Harvard Pilgrim Health Care Institute Human Studies Committee (IRB reference number 222927). The work was supported in part by funding from the Centers for Disease Control and Prevention.

## Results

Across all three seasons, there were 471 cases of severe ILI and 6127 cases of mild ILI. The median BMI was 27·8 among people with severe ILI, 27·3 for people with mild ILI, and 26·9 for patients without ILI. For all seasons combined, the crude OR for severe ILI among overweight and obese patients combined (BMI ≥ 25) was 1·45 (95% CI 1·18, 1·78). After adjusting for age, Charlson score, and sex, the OR for BMI ≥ 25 and severe ILI was 1·10 (95% CI 0·89, 1·37). For obese patients with BMI ≥ 30, the odds ratio for severe ILI was 1·65 (95% CI 1·31, 2·08). After adjusting for age, Charlson score, and sex, the OR for obesity and severe ILI was 1·23 (95% CI 0·97, 1·57). The crude OR for overweight and severe ILI was 1·29 (95% CI 1·02, 1·62); the adjusted OR for overweight and severe ILI was 0·99 (95% CI 0·78, 1·26).

The characteristics of cases and controls, by season, are compared in Table 2. Severe ILI cases were older compared with mild ILI cases and controls, although they were substantially younger during H1N1 (median 57 years) compared with the two regular seasons (median 72 years). The proportion of females among controls, mild cases, and severe cases varied by season. Sixty-eight percent (68%) of the hospitalized cases had a pneumonia-related diagnosis (i.e., general pneumonia ICD-9 code or organism-specific pneumonia code). By season, 79%, 57%, and 66% of severe cases were pneumonia-related in 2003–2004, 2004–2005, and H1N1, respectively.

**Table 2:** Characteristics of mild and severe cases of influenza-like illness (ILI) and controls for three influenza seasons (2003–2004; 2004–2005; 2009 H1N1) in Massachusetts

	Severe ILI	Mild ILI	Controls
2003–2004	*n* = 202	*n* = 2194	*n* = 8929
Age–Mean ± SD	68 ± 16	52 ± 18	51 ± 15
Age–Median	72	52	51
BMI–Mean ± SD	29 ± 6·3	28 ± 6·0	28 ± 5·7
BMI–Median	28	27	27
	No. (%)	No. (%)	No. (%)
Normal weight	58 (29)	745 (34)	3057 (34)
Overweight	71 (35)	757 (35)	3234 (36)
Obese	73 (36)	692 (32)	2638 (30)
Female	103 (51)	1514 (69)	5687 (64)
Charlson score			
0	81 (40)	1476 (67)	7064 (79)
1	32 (16)	404 (18)	1083 (12)
≥2	89 (44)	314 (14)	782 (8·8)
Vaccinated*	80 (40)	641 (29)	2202 (25)
Race			
White	12 (5·9)	364 (17)	1580 (18)
Black	5 (2·5)	93 (4·2)	324 (3·6)
Asian	1 (0·50)	24 (1·1)	81 (0·91)
Other	0	2 (<0·10)	9 (<0·10)
Unknown/missing	184 (91)	1711 (78)	6935 (78)
2004–2005	*n* = 204	*n* = 2614	*n* = 10 117
Age–Mean (SD)	69 ± 14	54 ± 17	52 ± 16
Age–Median	72	54	51
BMI–Mean (SD)	29 ± 6·4	29 ± 6·3	28 ± 5·7
BMI–Median	28	28	27
	No. (%)	No. (%)	No. (%)
Normal weight	53 (26)	803 (31)	3487 (34)
Overweight	77 (38)	891 (34)	3625 (36)
Obese	74 (36)	920 (35)	3005 (30)
Female	120 (59)	1719 (66)	6313 (62)
Charlson score			
0	77 (38)	1628 (62)	7686 (76)
1	45 (22)	564 (22)	1438 (14)
≥2	82 (40)	422 (16)	993 (9·8)
Vaccinated*	122 (60)	955 (37)	2663 (26)
Race			
White	14 (6·9)	464 (18)	1919 (19)
Black	8 (3·9)	123 (4·7)	451 (4·5)
Asian	1 (0·49)	16 (0·61)	135 (1·3)
Other	0	2 (<0·10)	7 (<0·10)
Unknown/missing	181 (89)	2009 (77)	7605 (75)
2009 H1N1	*n* = 65	*n* = 1319	*n* = 6882
Age–Mean (SD)	57 ± 11	46 ± 15	47 ± 13
Age–Median	57	48	49
BMI–Mean (SD)	30 ± 6·9	29 ± 6·3	28 ± 5·9
BMI–Median	30	27	27
	No. (%)	No. (%)	No. (%)
Normal weight	13 (20)	418 (32)	2310 (34)
Overweight	20 (31)	461 (35)	2471 (36)
Obese	32 (49)	440 (33)	2101 (31)
Female	32 (49)	885 (67)	4012 (58)
Charlson score			
0	27 (42)	913 (69)	5441 (79)
1	12 (18)	262 (20)	912 (13)
≥2	26 (40)	144 (11)	529 (7·8)
Vaccinated*	11 (16·9)	109 (8·3)	379 (5·5)
Race			
White	32 (49)	634 (48)	3327 (48)
Black	11 (17)	155 (12)	749 (11)
Asian	0	38 (2·9)	232 (3·4)
Other	0	3 (0·23)	16 (0·23)
Unknown/missing	22 (34)	489 (37)	2558 (37)

BMI, body mass index; SD, standard deviation; EHR, electronic health record.

Severe ILI includes only those hospitalized with ILI. This table contains data for all three influenza seasons.

* Vaccine data were only available for those who received influenza vaccine at an HVMA site (seasonal influenza vaccine transaction recorded in electronic health record). “Other” race contains American Indian/Alaskan Natives, Native Hawaiian, or Other Pacific Islander, and those with more than one race reported.

While it appears that severe ILI cases had a higher proportion of having received seasonal influenza vaccine compared with mild ILI cases and controls, the vaccination information is incomplete in this dataset because individuals may receive influenza vaccine outside of their primary care provider's office, and those doses would not be captured in the EHR. We therefore cannot interpret or analyze vaccination status. Approximately two-thirds of individuals had missing race information in the full dataset, and we therefore cannot interpret these data.

To evaluate severe ILI among otherwise healthy adults, we restricted the analysis to individuals with a Charlson score of zero. Among those with no identified comorbidities, the crude OR for obesity and severe ILI was 1·83 (95% CI 1·27, 2·65). After adjusting for age and sex, the OR was 1·55 (1·07, 2·25). The crude OR for overweight and severe ILI among those with a Charlson score of zero was 1·33 (0·92, 1·92); the adjusted OR was 1·07 (0·73, 1·55).

### BMI and ILI by Influenza Season

Table 3 contains results from logistic regression models examining the association between overweight and obesity with severe and mild ILI, by influenza season. There is evidence of a small association between obesity and severe ILI in 2003–2004 (OR 1·14, 95% CI 0·80, 1·64) and 2004–2005 (OR 1·24, 95% CI 0·86, 1·79), and a larger association for 2009 H1N1 (OR 1·76, 95% CI 0·91, 3·42), although not statistically significant at the *P* < 0·05 level. There does not appear to be an association between overweight and severe ILI for any of the three study periods.

**Table 3:** Crude and adjusted odds ratio (OR) and 95% confidence intervals (95% CI) for severe and mild influenza-like illness (ILI) by season

Influenza season	BMI category	Severe ILI		Mild ILI	
	
		Crude OR	Adjusted OR	Crude OR	Adjusted OR
2003–2004	Overweight	1·16 (0·82, 1·64)	0·88 (0·61, 1·26)	0·96 (0·86, 1·08)	0·99 (0·89, 1·12)
	Obese	1·46 (1·03, 2·07)	1·14 (0·80, 1·64)	1·08 (0·96, 1·21)	1·06 (0·94, 1·19)
2004–2005	Overweight	1·40 (0·98, 1·99)	1·11 (0·77, 1·59)	1·07 (0·96, 1·19)	1·06 (0·95, 1·18)
	Obese	1·62 (1·14, 2·31)	1·24 (0·86, 1·79)	1·33 (1·20, 1·48)	1·26 (1·13, 1·40)
2009 H1N1	Overweight	1·44 (0·71, 2·90)	1·09 (0·53, 2·24)	1·03 (0·89, 1·19)	1·17 (1·00, 1·35)
	Obese	2·71 (1·42, 5·17)	1·76 (0·91, 3·42)	1·16 (1·00, 1·34)	1·22 (1·05, 1·42)

BMI, body mass index.

Severe ILI is a hospitalized ILI visit. Mild ILI is an outpatient ILI visit. Reference group is normal weight (BMI 18·5–24·9). Adjusted for sex, age (continuous), and Charlson Comorbidity Index score (continuous).

Our results suggest there may be a small association between obesity and mild ILI in 2004–2005 (OR 1·26, 95% CI 1·13, 1·40) and 2009 H1N1 (OR 1·22, 95% CI 1·05, 1·42). The association between overweight and mild ILI is small or close to null for both of those periods. Table 4 includes results from the same analyses as above, restricted to individuals with a Charlson score of zero. The results are generally consistent, although the ORs are larger and the confidence intervals wider due to fewer cases.

**Table 4:** Crude and adjusted odds ratio (OR) and 95% confidence intervals (95% CI) for severe and mild influenza-like illness (ILI) by season, among those with a Charlson Comorbidity Index score of zero (0)

Influenza season	BMI category	Severe ILI		Mild ILI	
	
		Crude OR	Adjusted OR	Crude OR	Adjusted OR
2003–2004	Overweight	1·12 (0·64, 1·97)	0·90 (0·51, 1·59)	0·92 (0·81, 1·05)	0·98 (0·85, 1·12)
	Obese	1·91 (1·11, 3·27)	1·60 (0·93, 2·76)	1·01 (0·88, 1·16)	1·05 (0·91, 1·21)
2004–2005	Overweight	1·43 (0·82, 2·48)	1·12 (0·64, 1·97)	1·07 (0·94, 1·21)	1·09 (0·96, 1·25)
	Obese	1·65 (0·93, 2·93)	1·43 (0·80, 2·56)	1·30 (1·14, 1·49)	1·33 (1·16, 1·52)
2009 H1N1	Overweight	1·81 (0·67, 4·91)	1·54 (0·56, 4·27)	0·98 (0·83, 1·16)	1·14 (0·96, 1·35)
	Obese	2·26 (0·82, 6·23)	1·90 (0·68, 5·27)	1·09 (0·91, 1·29)	1·23 (1·03, 1·47)

BMI, body mass index.

Severe ILI is a hospitalized ILI visit. Mild ILI is an outpatient ILI visit. Reference group is normal weight (BMI 18·5–24·9); overweight = BMI 25·0–29·9; obese = BMI ≥ 30. Adjusted for sex and age (continuous).

### BMI and ILI by Age Group

For 20- to 39-year-olds, the median BMI among severe cases (*n* = 24), mild cases (*n* = 1575), and controls (*n* = 6256) was 26·8, 25·9, and 25·4, respectively. For 40- to 59-year-olds, the median BMI among severe cases (*n* = 118), mild cases (*n* = 2572), and controls (*n* = 13 046) was 29·4, 27·9, and 27·2, respectively. For those aged 60 and older, the median BMI among severe cases (*n* = 329), mild cases (*n* = 1980), and controls (*n* = 6626) was 27·4, 27·7, and 27·6. The adjusted OR for those aged 20–59 years was 1·92 (95% CI 1·26, 2·90) and was 1·08 (95% CI 0·80, 1·46) for those 60 and older. When we further stratified the younger age group (Table 5), there appears to be an association that does not achieve statistical significance at the *P* < 0·05 level, between obesity and severe ILI among 20- to 39-year-olds (OR 2·43, 95% CI 0·91, 6·46); and a statistically significant association is observed for 40- to 59-year-olds (OR 1·84, 95% CI 1·16, 2·19). There is no evidence of an association between overweight and severe ILI in any of the three age groups. When we restricted the age group analyses to those with a Charlson score of zero (Table 6), the results were generally very similar.

**Table 5:** Crude and adjusted odds ratio (OR) and 95% confidence intervals (95% CI) for severe and mild influenza-like illness (ILI) by age group

Age group	BMI category	Severe ILI		Mild ILI	
	
		Crude OR	Adjusted OR	Crude OR	Adjusted OR
20–39 years	Overweight	1·30 (0·47, 3·58)	1·47 (0·52, 4·15)	1·05 (0·93, 1·20)	1·18 (1·04, 1·35)
	Obese	2·33 (0·90, 6·06)	2·43 (0·91, 6·46)	1·21 (1·05, 1·38)	1·33 (1·16, 1·54)
40–59 years	Overweight	1·04 (0·63, 1·72)	1·03 (0·62, 1·72)	1·03 (0·93, 1·14)	1·10 (0·98, 1·22)
	Obese	2·05 (1·30, 3·23)	1·84 (1·16, 2·19)	1·31 (1·18, 1·46)	1·31 (1·17, 1·45)
≥60 years	Overweight	0·98 (0·74, 1·30)	0·99 (0·75, 1·33)	0·95 (0·84, 1·08)	0·99 (0·87, 1·12)
	Obese	1·04 (0·78, 1·38)	1·08 (0·80, 1·46)	1·03 (0·91, 1·18)	1·04 (0·91, 1·19)

BMI, body mass index.

Severe ILI is a hospitalized ILI visit. Mild ILI is an outpatient ILI visit. Reference group is normal weight (BMI 18·5–24·9); overweight = BMI 25·0–29·9; obese = BMI ≥ 30. Adjusted for sex, age (continuous), and Charlson Comorbidity Index score (continuous).

**Table 6:** Crude and adjusted odds ratio (OR) and 95% confidence intervals (95% CI) for severe and mild influenza-like illness (ILI) by age group, among those with a Charlson Comorbidity Index score of zero (0)

Age group	BMI category		Severe ILI		Mild ILI	
	
			Crude OR	Adjusted OR	Crude OR	Adjusted OR
20–39 years	Overweight		0·76 (0·23, 2·51)	0·95 (0·28, 3·22)	1·02 (0·89, 1·17)	1·15 (0·99, 1·33)
	Obese		1·94 (0·70, 5·35)	2·30 (0·82, 6·51)	1·21 (1·04, 1·40)	1·35 (1·16, 1·58)
40–59 years	Overweight		1·58 (0·77, 3·21)	1·51 (0·73, 3·12)	1·03 (0·91, 1·16)	1·08 (0·95, 1·23)
	Obese		2·67 (1·35, 5·28)	2·61 (1·31, 5·19)	1·22 (1·07, 1·38)	1·26 (1·11, 1·43)
≥60 years		Overweight	0·99 (0·62, 1·59)	1·02 (0·63, 1·64)	0·98 (0·82, 1·16)	1·01 (0·85, 1·20)
		Obese	1·14 (0·70, 1·88)	1·26 (0·76, 2·09)	1·06 (0·88, 1·28)	1·10 (0·91, 1·32)

BMI, body mass index.

Severe ILI is a hospitalized ILI visit. Mild ILI is an outpatient ILI visit. Reference group is normal weight (BMI 18·5–24·9); overweight = BMI 25·0–29·9; obese = BMI ≥ 30. Adjusted for sex and age (continuous).

Finally, we conducted a preliminary analysis adjusting for race, among the small proportion of individuals with complete race information in the dataset. For H1N1 (total *n* = 5197; 43 severe ILI cases), after adjusting for race, sex, age, and Charlson score, there was clear indication of an association between obesity and severe ILI (OR 4·68, 95% CI 1·58, 13·85) and between overweight and severe ILI (OR 3·07, 95% CI 0·99, 9·43). For the two regular seasons, there were very few severe ILI cases among those with race information (∼20 per season) so the confidence intervals were very wide and the results difficult to interpret.

## Discussion

This study provides evidence of a small to moderate association between obesity and hospitalized ILI, the size of which appears to vary by influenza season and age group. These results support previous findings of an association between obesity and severe ILI identified during the H1N1 influenza A pandemic, but we also identified evidence of obesity as an independent risk factor for hospitalized ILI during non-pandemic influenza seasons. Importantly, when restricted to individuals without comorbidities (i.e., a Charlson Comorbidity Index score of zero), the association was still apparent and the odds ratios were larger. Additionally, our results suggest there may be a modest association between obesity and outpatient ILI. The relation between overweight and hospitalized ILI in this study is less clear and, if present, may be small. While not all of the study results reached statistical significance, they suggest that obesity may be an independent risk factor of severe ILI, particularly among healthy, young adults, given the consistent, positive direction of our findings.

For severe ILI, our results are comparable to, although more modest than, those reported by Kwong *et al*.^11^ Those authors examined the association between self-reported BMI and respiratory hospitalizations over 12 influenza seasons in Ontario (*n* > 82 000). Among those without a risk factor for influenza complications, those with a BMI of 30–34·9 had an OR of 1·73 (95% CI 0·86, 3·46) compared with normal weight individuals, and those with a BMI ≥ 35 had an OR of 5·10 (95% CI 2·53, 10·24). When the authors stratified their analyses by the number of risk factors, the ORs and corresponding confidence intervals were smaller. Another group examined BMI and outpatient laboratory-confirmed influenza for two regular seasons (2007–2008 and 2008–2009) and 2009 H1N1.^10^ For 2009 H1N1, they reported small increased odds of outpatient influenza among overweight (OR 1·40, 95% CI 0·80, 2·45) and obese (BMI 30–39·9: OR 1·02, 95% CI 0·59, 1·78; BMI ≥ 40: OR 1·53, 95% CI 0·76, 3·08) compared with normal weight. The results for the two non-pandemic periods are generally suggestive of a null association.

The study described here contributes to a small but growing literature on the relation between weight, and ILI occurrence and outcome. Strengths of the study include the large sample size and use of professionally ascertained weight and height for BMI calculation rather than patient self-reports. The robust EHR used by HVMA, and the practice's consistent use of practice-wide guidelines for situations such as managing ILI during respiratory illness seasons allow for confidence in the stability of the dataset. The data quality is good insofar as it is a paperless practice that has been using EHRs for over 20 years. The EHR is used for all clinical transactions and is therefore deeply engrained into routine practice. The limitations of the dataset are those common to all retrospective observational studies: The data are limited to patients that opted to seek medical care and contingent upon different clinician's diagnostic, testing, and coding choices, all of which were made primarily for the purposes of immediate patient care rather than for surveillance research. To the extent that clinicians did assign diagnoses to patients, however, the EHR is likely to be comprehensive. Because the diagnosis codes for case determination were drawn from patients' insurance claims records, they include data from encounters outside the primary study practice (e.g. specialty referrals and hospitalizations). They are therefore likely to be complete. It is also important to note that the cohorts were restricted to individuals without a hospitalization in the prior year, meaning that the study was among healthier individuals than the larger patient population.

The study has several limitations. We examined ILI derived from ICD-9 codes and were not able to examine laboratory-confirmed influenza. Our syndromic definition captures respiratory illness due to multiple pathogens in addition to influenza. We originally planned to restrict the analysis to the periods within each season when influenza activity was at its peak, but applying the ILI definition to a small number of weeks per season yielded very few hospitalized cases. While the HVMA EHR is a well-established system, information gaps remain. Completeness and accuracy of smoking status and influenza vaccine exposure in the EHR were too unreliable to analyze. While we do not believe these covariates are confounders, they may be modifiers. For example, higher BMI has been associated with less frequent influenza vaccination among the elderly.^18^ Data on race and ethnicity were missing for over 70% of cases and controls in the regular influenza seasons and for 37% during 2009 H1N1. The proportion of individuals who were overweight or obese may vary substantially by racial group. Among those with documented race, 43% of Asians were overweight or obese, 63% of Whites were overweight or obese, 75% of those identified as “other” were overweight or obese, and 83% of Blacks were overweight or obese. Among those with missing race information, 67% were overweight or obese. In a separate (unpublished) analysis of ILI in Massachusetts, some of this study's co-authors found substantial disparities in rates of ILI hospitalization by race during seasonal and pandemic years. We therefore have concerns about potential residual confounding due to missing race and ethnicity data, as well as other covariates related to socioeconomic status. Our preliminary analysis for H1N1, among those with race information, resulted in a larger association for both obesity and overweight with severe ILI, suggesting that race and ethnicity may be important covariates. Additionally, the study was restricted to those with a documented BMI. We have data available on the frequency and characteristics of patients with missing BMIs for the H1N1 study period, but unfortunately not for the other seasons. For the H1N1 period, of the 35 099 individuals originally available for analysis, only 6% (*n* = 2179) did not have BMI recorded within 12 months of the study period start date (and were therefore excluded from our full analysis). We compared those with and without BMI recorded with respect to gender, age, and Charlson score. Men were more likely to be missing BMI than females (10% compared with 3·6%). The average age of those with a measured BMI was 51 compared with an average age of 43 for those without BMI. Seventy-five percent of those with a recorded BMI had a Charlson score of zero compared with 91% among those without a BMI recorded. These differences, while important, are not surprising as one may assume that males, younger adults, and those without comorbidities would be less likely to have annual medical visits. Finally, because the Charlson Comorbidity Index does not capture certain comorbidities associated with increased risk of severe influenza or other respiratory infections (e.g., asthma not identified as chronic lung disease), those with a score of zero may actually have an underlying illness that put them at increased risk of severe ILI. These comorbidities may also be associated with BMI, so raise an additional concern about residual confounding.

## Conclusions

Our study provides support for an association between obesity and hospitalized ILI, varying by age group and influenza season. A similar, but possibly smaller association, may exist for obesity and outpatient ILI. These effects were enhanced when limiting the analysis to patients without known comorbidities, suggesting that even so-called “healthy” overweight and obese adults may remain at increased risk of ILI requiring hospitalization. Directions for future research include larger studies that can capture larger numbers of cases, studies designed to target subgroups (e.g., younger ages), and studies designed to collect complete and accurate data on covariates such as race, ethnicity, comorbidities, and vaccination status.
